# Burden and Associated Genotype Patterns of High-Risk Human Papilloma Virus Infection and Cervical Cytology Abnormalities among Women in Central India

**DOI:** 10.1155/2022/3932110

**Published:** 2022-05-18

**Authors:** Shipra Gupta, Shashank Purwar, Priyal Gupta, Ajay Halder, Ayush Gupta, K. Pushpalatha, Julie Hansa John

**Affiliations:** ^1^Department of Microbiology, AIIMS, Bhopal, India; ^2^Department of OBGY, AIIMS, Bhopal, India

## Abstract

**Background:**

The epidemiology of human papilloma virus (HPV) infection and the pattern of HPV genotype distribution are much-needed parameters to assess the risk of cervical cancer among females. However, due to less availability of data on HPV burden and its genotypes from various geographical regions in India makes cervical cancer screening modalities and vaccination strategies difficult to implement.

**Objective:**

The present study was conducted to identify the various genotypes particularly high-risk HPV types in premalignant or malignant cervical lesions.

**Methods:**

The study was a hospital-based cross-sectional study wherein 295 symptomatic women were screened by Pap smear and multiplex real-time PCR was performed for HPV genotypes identification in women with abnormal cervical cytology.

**Results:**

Out of 295 women, 237 (80.3%), 45 (15.3%), and 13 (4.4%) women had normal Pap smear, squamous cell carcinoma and precancerous cytology, respectively. Among these 58 women having abnormal cervical cytology, HPV was detected in 48 (81.0%) participants. Most common HPV genotypes in our study were HPV 16 (*n* = 29; 60.4%) followed by mixed infections; i.e., more than one type of HPV was detected (*n* = 10, 20.8%). HPV 18 was detected only in 6.25%, whereas other high-risk HPV genotypes were found to be 12.5%.

**Conclusion:**

HPV positivity was >80% in women having abnormal Pap smear. The prevalence of HPV 18 was found to be much less in Central India, compared to other parts of country. HPV 16 was the most common genotype followed by mixed HPV genotype infections. It is evident from our study that symptomatic women even if having normal Pap smear should be screened for HPV and followed up with periodic Pap smears for detecting any change in cervical cytology, thus preventing cervical cancer in women.

## 1. Introduction

Cervical cancer is the second most common cancer among women worldwide [1]. In India, the total number of cervical cancer cases in 2020 was 123,907 accounting for 18.3% of all cancer cases among women while deaths were 77,348 accounting for 18.7% of female cancer deaths [2]. Human papillomavirus (HPV) is one of the most common causes of anogenital cancers in both men and women worldwide. Approximately 40 HPV genotypes are linked to anogenital infections, and they are divided into three categories based on their oncogenic potential: high, low, and intermediate risk types. HPV 16, 18, 31, 33, 35, 39, 45, 51, 52, 56, 58, 59, 68, 73, and 82 are of high-risk or oncogenic types due to their presence in high-grade squamous intraepithelial infection (HSIL) or cervical cancer, while HPV 6, 11, 40, 42, 43, 44, 54, 61, 70, 72, and 81 are referred to as low-grade types and are associated with benign warts and HPV 23, 53, and 66 as intermediate risk types [3].

A persistent infection with a high-risk HPV contributes to the development of invasive cervical cancer. In rural India, due to poor hygiene, lack of awareness, screening, and vaccination, HPV infection largely goes undetermined. Therefore, in India, the frequency of cervical cancer is relatively high. Various studies conducted in different geographical regions in India reported wide variations in the frequency of HPV infection and genotype distribution, but still there is no countrywide data on HPV infection and genotype distribution, which could be useful for implementation of screening modalities and vaccination strategies [4].

Less than 5% of women in developing countries undergo Pap smear screening due to lack of effective, organized, and opportunistic cervical cytology screening programs anywhere in the country [5]. The epidemiology of HPV infection and the pattern of HPV genotype distribution, in Central India, are largely unexplored. Therefore, the aim of this study is to determine the HPV burden and its genotype distribution in women with abnormal cervical cytology.

## 2. Material and Methods

### 2.1. Study Design, Settings, and Participants

It was a hospital-based cross-sectional study conducted in departments of Microbiology, Obstetrics and Gynaecology and Pathology at AIIMS, Bhopal. Women between 30 and 65 years of age presenting with symptoms like abnormal vaginal bleeding/discharge, pain during coitus, and lower abdominal pain were examined using PAP smear. The patients with abnormal PAP smear and women showing growth on speculum examination were included in the study after obtaining the consent. Patients not willing to give consent and treated cases of cervical cancer and patients undergoing chemotherapy or radiotherapy and conditions contraindicated for PAP smear testing-hysterectomy were excluded from the study.

### 2.2. Sample Collection and Processing

For women who satisfied the inclusion criteria and gave consent to participate in the study, their sample for HPV testing was taken during gynaecological examination. The cervix was visualized using a Cusco's speculum. Complete visualization of external os with squamocolumnar junction was ensured for adequate sampling. Ayer's spatula was placed on the external os and rotated unidirectionally through 360 degrees. The specimen was then smeared onto the glass slide which was placed in Coplin's jar containing 95% ethanol solution. The cytological classification was done according to the Bethesda System.

To avoid frequent follow-up by the patient, for suspicious cases, cervical washings were taken in DNA LBC cervical sample transport medium according to the manufacturer's protocol (3B Blackbio Biotech India Ltd.) along with the PAP smear. Collected samples were stored at 4°C till further processing. Since PAP smear was not always possible mostly in cases of invasive cancer, therefore, histopathology reports were also used for confirmation of cervical cancer.

### 2.3. DNA Extraction and HPV Genotype Detection

DNA extraction for 58 samples was done using QIAamp DNA mini kit as per the manufacturer's instructions. TRUPCR® HPV HR with 16/18 Genotyping Kit was used for detection and genotyping of Human papilloma virus DNA in clinical samples. This kit used fluorescent reporter dye probes specific for detection of high risk HPV 14 genotypes (16, 18, 31, 33, 35, 39, 45, 51, 52, 56, 58, 59, 66, and 68) with genotyping of 16 and 18. In this kit, there are three independent reactions running parallel in three tubes; the first detects any of the HPV HR genotypes 16, 31, 33, 35, 52, 58, 51, 56, and 66 (FAM channel); the second detects any of the HPV HR genotypes 18, 45, and 59 (FAM channel) along with genotyping of HPV 18 (HEX) and endogenous internal control (IC) (TEXAS RED) which allows excluding unreliable results and third detects either HPV HR genotype 39 or 68 (FAM) along with genotyping of HPV 16 (TEXAS RED).

### 2.4. Statistical Analysis

Data were analysed and statistically evaluated using SPSS software, version 25 (Chicago II, USA). Quantitative data was expressed in mean and standard deviation while qualitative data were expressed in percentage.

### 2.5. Ethical Issues

The study was approved by the Institutional Human Ethical Committee. All participants were explained about the purpose of the study. Confidentiality was assured to them along with informed written consent.

### 2.6. Observation and Results

**Figure 1:**
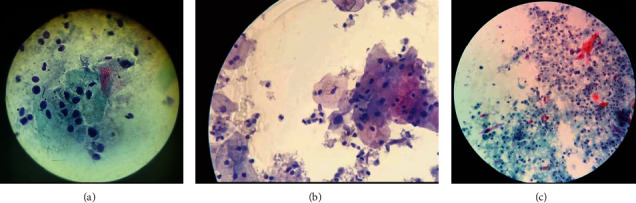
(a) NILM. (b) Precancerous lesions. (c) Cancerous lesions. ^∗^NILM: negative for intraepithelial lesion/malignancy.

Total 295 women were screened using PAP smear out of which 237 were found to be NILM (Figure 1(a)), while 58 had abnormal PAP result, of which 13 (4.4%) were found to be precancerous (Figure 1(b)) and 45 (15.3%) were found to be of cancerous type (Figure 1(c)).

**Figure 2:**
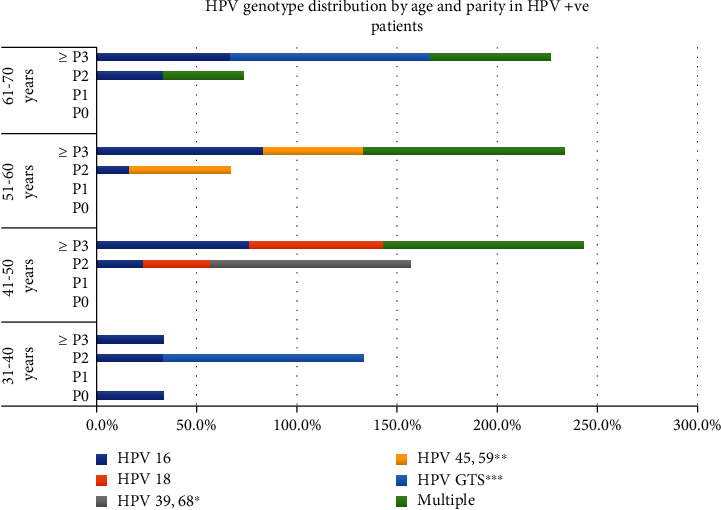
HPV genotype distribution by age and parity in HPV-positive patients.

These 58 study participants were further tested for Human papilloma virus (HPV). Among them, HPV was detected in 48 (81.0%) participants.  Table 1 shows age and parity wise distribution of HPV-positive patients which is graphically represented in Figure 2. Out of 48 patients who were found to be HPV-positive, 4 (8.3%) were in the age group of 31-40 years, 24 (50%) were in the age group of 41-50 years, and 10 (20.8%) were in the age group of 51-60 years while 10 (20.8%) were >60 years old. The highest HPV positivity was seen in the age group of 41-50 years 24 (50%). In terms of parity distribution, 34 (70.8%) women had parity 3 or more.

The most common HPV type in our study was HPV 16 (*n* = 29; 60.4%) followed by mixed HPV infection (*n* = 10, 20.8%). Other genotypes of HPV found were HPV18 (*n* = 3; 6.25%), HPV GTS∗∗∗ (*n* = 3; 6.25%), HPV 45 or 59∗∗ (*n* = 2; 4.2%), and HPV 39 or 68∗ (*n* = 1; 2.1%) as shown in Table 2.

**Figure 3:**
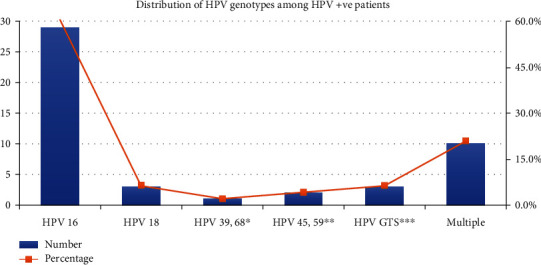
Distribution of HPV genotypes among HPV-positive patients.

**Figure 4:**
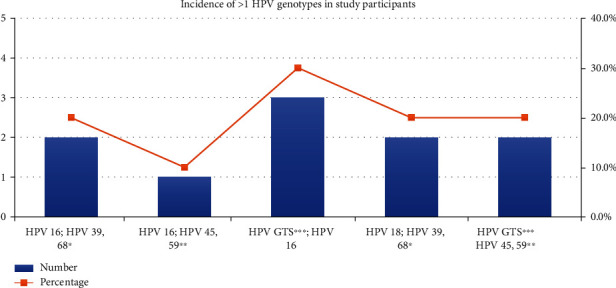
Distribution of mixed HPV genotypes in study participants.

Distribution of various HPV genotypes was detected in single (Figure 3) and mixed infections (Figure 4).

The most common histological type among HPV-positive patients was found to be of squamous cell carcinoma (*n* = 41, 97.6%). Mucinous adenocarcinoma was found in only 1 (2.4%) patient (Table 3).

Among the HPV-positive squamous cell carcinoma (type unknown) cases (*n* = 21), HPV 16 was the most common genotype (66.7%) followed by mixed genotypes (19.0%), HPV GTS∗∗∗ (9.5%), and HPV 18 (4.8%). Among HPV-positive nonkeratinizing SCC, HPV 16 genotype was detected in 7 (50.0%) samples and multiple in 5 (35.7%), and HPV 45, 59 was detected in 2 (14.3%) samples.

**Figure 5:**
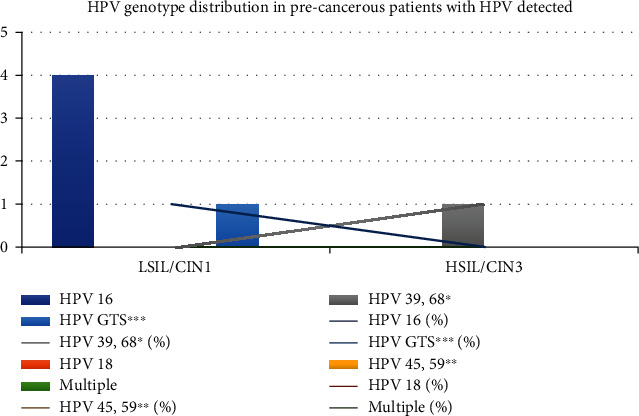
HPV genotype distribution in precancerous patients with HPV detected.

Of the HSIL lesions, HPV 16 was the most common genotype seen in 4 (80.0%) samples while in 1 sample HPV GTS∗∗∗ genotype was detected. There was only 1 LSIL lesion in which HPV 39/68 genotype was detected (Figure 5).

## 3. Discussion

The role of HPV infections in the development of cervical cancer has acquired fundamental importance, and in recent years, HPV testing in addition to PAP screening has become a relevant diagnostic and prognostic tool. The development of HPV vaccines holds tremendous promise for developing countries like India where cervical cancer is the most common malignancy among middle aged women [6]. Despite HPV vaccination, screening is considered a public health strategy to assess the impact of HPV vaccination, risk stratification of women, and the improvement of posttreatment surveillance. Therefore, it is important to understand the burden and distribution of the major HPV genotypes in various geographical regions.

Of the 295 patients screened using PAP smear, 58 (19.6%) of the participants had abnormal cytology. Of these, 13 (4.4%) and 45 (15.3%) participants were found to be precancerous and cancerous type, respectively. Among 13 patients, 7 (53.8%) and 6 (46.2%) were found to be LSIL and HSIL, respectively, which are in concordance with Maheshwari et al. [7] in which 384 women with high risk of cervical carcinoma were screened, among which 61 (15.89%) had abnormal cytology. The most common being LSIL contributing to about 11.2% followed by ASCUS (1.82%) and AGC (1.04%) with almost similar percentage of HSIL (0.78%) and AGUS and ASC-H (0.52% each).

58 study participants were further tested for Human papilloma virus (HPV). Among these 48 (81.0%) participants came positive for HPV infection. High risk HPV prevalence was approximately 93.3% in participants with invasive cervical cancer whereas 46.1% in participants with precancerous lesions, suggesting HPV as a major risk factor for cervical cancer. Studies from various geographical regions in India also suggested similar high risk HPV prevalence in patients with cervical cancer with approximately 87.8% in Andhra Pradesh by Sowjanya et al. [8] 99.4% in Chennai by Franceschi et al. [9]; however, in a study by Maheshwari et al. [7]. HPV DNA prevalence was found to be only 40.98% which was very low. This convincingly proves HPV as the “necessary” cause of cervical cancer. A meta-analysis of the worldwide prevalence of HPV in invasive cervical cancer by Clifford et al. [10] noted that 11-17% of cervical cancers have been reported to be HPV-negative whereas our study reported approximately 7% and 53.8% of cervical cancer and precancerous cases to be HPV-negative, respectively. Various studies have already suggested that cervical cancer often diagnosed at an advanced stage could be HPV-negative; however, in our study, we observed 2 participants who were HPV-negative were found to be in early stage of disease. In fact, LSIL cases were found to be more HPV-negative than HSIL cases. This could be due to latent HPV infection, nonhigh risk HPV infection, false negative test results, or absence of cancer cells in the sample.

Despite the fact that most of the infections due to HPV are resolved spontaneously, however, we observed, out of 48 patients who were found HPV positive with abnormal cytology, the highest HPV positivity (24 (50%)) was seen in the age group of 41-50 years. Similar results were reported by Sarma et al. [11] in Guwahati with increased prevalence of HPV infection (21.4%) in females more than 50 years of age.

It was also observed that parity of 2 or more is an important risk factor. This may be due to early age of marriages in semiurban India resulting in early child bearing. In terms of parity distribution, 34 (70.8%) women who were HPV positive with abnormal cytology had parity 3 or more. Kennedy et al. [12] found that HPV infection was higher in females with a parity of 3 or more. In Molina-Pineda et al.'s [13] study, the average number of pregnancies in the cervical cancer group (4.4 ± 3.3) was twice that observed in the CIN 1 group (2.1 ± 2.0). Different reports have shown that parity ≥ 3 is correlated with the risk of cervical cancer development.

The most common HPV type in our study was HPV 16 (*n* = 29; 60.4%) followed by multiple types (*n* = 10, 20.8%). Other genotypes of HPV found were HPV18 (*n* = 3; 6.25%), HPV 9 GTS∗∗∗ (*n* = 3; 6.25%), HPV 45 or 59 (*n* = 2; 4.2%), and HPV 39 or 68 (*n* = 1; 2.1%). The distribution of HPV types found in our study is quite similar to a recent large-scale study reported from India and is also consistent with the most common types found in South-East Asia [9, 14–16]. In a study by Sowjanya et al. [8], HPV 16 was the most prevalent type detected (24/36, 66.7%), followed by HPV 18 (7/36 19.4%); however, we observed comparatively less burden of HPV 18 in Central India. A similar study from Delhi reported that HPV 16 and 18 contributed to 73.6 and 14.2% cases of cervical carcinoma [17].

In our study, in participants with HPV infection, single infection was found in 79.2% of the cases while in 20.8% of the cases, multiple infections were seen. Our findings were similar to Maheshwari et al.'s [7] study in which 85.71% of the total tested cases were positive for only a single high-risk HPV type while the remaining samples had multiple combinations of HPV types with genotypes 16 and 18 and 16 and 31 being the most common (5.36% each). Other combinations seen were genotypes 16, 31, 33, and 35 and 31, 33, 35, and 39. Molina-Pineda et al. [13] concerning HPV coinfections determined that 75.7% (*n* = 56) were observed as single infection and 24.3% (*n* = 18) as multiple infection. In a multicentre study by Franceschi et al. [9] type 16 was most common genotype detected alone in 165/278 (59.4%) cases and in association with type 18 in 10 (3.6%) cases. Senapati et al. [4] reported prevalence of single and multiple genotypes was 76.58% and 23.41%, respectively.

In the present study, the most common histological type in cancerous lesion was found to be squamous cell carcinoma (*n* = 44, 97.8%). Mucinous adenocarcinoma was found in only 1 (2.2%) patient. Among the HPV-positive squamous cell carcinoma (type unknown) cases (*n* = 21), HPV 16 was the most common genotype (66.7%) followed by multiple genotypes (19.0%), HPV GTS∗∗∗ (9.5%), and HPV 18 (4.8%). Among HPV-positive nonkeratinizing SCC, HPV 16 genotype was detected in 7 (50.0%) samples and multiple in 5 (35.7%), and HPV 45, 59 was detected in 2 (14.3%) samples. In a study by Basu et al. [18], out of the 262 cases of SCC, HPV was detected in 243 (92.7%) and genotypes 16, 18, 31, 33, or 45, alone or in combination with each other, were the most common types accounting for 85.5% (224/262). HPV was detected in 9 of the 11 (81.8%) adenocarcinoma cases, and the same five genotypes accounted for all positive cases. Regardless, genotype 16 was the most common type, detected alone or in combination with other types, in both SCC and ADC, accounting for 66.4% (174/262) and 54.5% (6/11), respectively. In contrast to our study, HPV 16, HPV 18, and HPV 33 were detected in 64.8%, 14.7%, and 6.4%, respectively, in 423 cases of SCC reported in a meta-analysis of Indian studies by Bhatla et al. [19]. Srivastava et al. [3] also reported squamous cell carcinoma as the most common histological type reported in 94.8% cases while adenocarcinoma in the rest of 5.2% cases. In their study, HPV 16 was present in 71.7% (*n* = 66) of the SCC cases followed by HPV 18 (*n* = 23, 25%) and HPV 31 (*n* = 14, 15.2%).

In our study, among HSIL lesions, HPV 16 was the most common genotype seen in 4 (80.0%) samples while in 1 sample HPV GTS∗∗∗ genotype was detected. There was only 1 LSIL lesion in which HPV 39/68 genotype was detected. Our findings were in concordance with Sarma et al. [11], where HSIL was seen in lesions in women who were infected with HPV genotype 16 and 18. Bakir et al. [20], from Turkey, found that in HSIL lesion HPV16 and HPV 51 were the genotype detected while in LSIL lesion multiple genotypes were detected (HPV 31/51, HPV 16/52, HPV 56/58, and HPV 16/52/66/68).

The HPV genotyping kit could identify HPV genotypes 16 and 18, but for other high risk genotypes, it could only detect the genotypes in combination (e.g., HPV 39, 68∗) but could not provide a definite single genotype identification.

## 4. Conclusion and Recommendations

It can be concluded from the study that HPV was detected in 93.3% of the cancerous patients and 46.2% of precancerous patients, confirming HPV as an important risk factor in disease progression. We identified HPV 16 as the most prevalent HPV type. Various other high-risk genotypes were also identified in our study, which offers the baseline data for future research and postvaccination surveillance.

## Figures and Tables

**Table 1:** Age-wise parity distribution of HPV-positive patients (*n* = 48).

	HPV type						
	HPV 16	HPV 18	HPV 39, 68	HPV 45, 59	HPV 9 GTS	Multiple	Total (*n* = 48)
*Age in years*							
31-40 years	3 (75%)	0 (0.0%)	0 (0.0%)	0 (0.0%)	1 (25%)	0 (0.0%)	4 (8.3%)
41-50 years	17 (70.8%)	3 (12.5%)	1 (4.2%)	0 (0.0%)	0 (0.0%)	3 (12.5%)	24 (50.0%)
51-60 years	6 (60%)	0 (0.0%)	0 (0.0%)	2 (20%)	0 (0.0%)	2 (20.0%)	10 (20.8%)
61-70 years	3 (30%)	0 (0.0%)	0 (0.0%)	0 (0.0%)	2 (20%)	5 (50%)	10 (20.8%)
*Parity*							
P0	1 (100.0%)	0	0	0	0	0	1 (2.1%)
P2	7 (53.8%)	1 (7.7%)	1 (7.7%)	1 (7.7%)	1 (7.7%)	2 (15.4%)	13 (27.1%)
≥P3	21 (61.7%)	2 (5.9%)	0	1 (2.9%)	2 (5.9%)	8 (23.5%)	34 (70.8%)

^∗^Maximum age was 65, applicable to all age analyses in this study.

**Table 2:** Distribution of HPV genotypes among HPV-positive patients (*n* = 48).

HPV type	Frequency	Percentage
*Single infection (n* = 38*; 79.2%)*		
HPV 16	29	60.4%
HPV 18	3	6.25%
HPV 39, 68^∗^	1	2.1%
HPV 45, 59^∗∗^	2	4.2%
HPV GTS^∗∗∗^	3	6.25%
Mixed	10	20.8%
*Mixed infection (n* = 10*; 20.8%)*		
HPV 16; HPV 39, 68^∗^	2	20.0%
HPV 16; HPV 45, 59^∗∗^	1	10.0%
HPV GTS^∗∗∗^; HPV 16	3	30.0%
HPV 18; HPV 39, 68^∗^	2	20.0%
HPV GTS^∗∗∗^; HPV 45, 59^∗∗^	2	20.0%

^∗^HPV genotype could be either 39 or 68. ^∗∗^HPV genotype could be either 45 or 59. ^∗∗∗^HPV genotype could be any of 31, 33, 35, 51, 52, 55, 56, 58, and 66 genotypes.

**Table 3:** HPV genotype distribution in cancerous and precancerous patients with HPV detected (*n* = 48).

Histological type	HPV 16	HPV 18	HPV 39, 68	HPV 45, 59	HPV 9 GTS	Multiple
SCC (type unknown)	14 (66.7%)	1 (4.76%)	0 (0.0%)	0 (0.0%)	2 (9.5%)	4 (19.0%)
Nonkeratinizing SCC	7 (50.0%)	0 (0.0%)	0 (0.0%)	2 (14.3%)	0 (0.0%)	5 (35.7%)
Keratinizing SCC	4 (66.7%)	1 (16.7%)	0 (0.0%)	0 (0.0%)	0 (0.0%)	1 (16.7%)
Mucinous adenocarcinoma	0 (0.0%)	1 (100.0%)	0 (0.0%)	0 (0.0%)	0 (0.0%)	0 (0.0%)
*Histological differentiation of cancerous lesion*						
Well differentiated	14 (56.0%)	2 (8.0%)	0 (0.0%)	1 (4.0%)	2 (8.0%)	6 (24.0%)
Moderately differentiated	11 (68.7%)	0 (0.0%)	0 (0.0%)	1 (6.2%)	0 (0.0%)	4 (25.0%)
Poorly differentiated	0 (0.0%)	1 (100.0%)	0 (0.0%)	0 (0.0%)	0 (0.0%)	0 (0.0%)
*Precancerous lesion*						
HSIL/CIN3	4 (80.0%)	0	0	0	1 (20.0%)	0
LSIL/CIN1	0	0	1 (100.0%)	0	0	0

## Data Availability

Descriptive data and analysis, as required, are included in the attached manuscript body (doc file).
